# Editorial: Gene therapy and genome editing for metabolic liver disorders

**DOI:** 10.3389/fmmed.2024.1543685

**Published:** 2025-01-09

**Authors:** Fanny Collaud, Giulia Bortolussi

**Affiliations:** ^1^ Genethon, Evry, France; ^2^ Université Paris-Saclay, Univ Evry, Inserm, Genethon, Integrare Research Unit UMR_S951, Evry, France; ^3^ International Centre for Genetic Engineering and Biotechnology, Trieste, Italy

**Keywords:** gene therapy, genome editing, metabolic liver disorders (MLD), rare genetic diseases, translational research

Metabolic liver disorders are a diverse group of conditions that affect the ability of the liver to process and metabolize nutrients, which can lead to severe complications if left untreated, such as liver damage or even liver failure. These disorders are often the result of genetic mutations affecting enzymes, transporters, structural proteins, or organelles that are essential for metabolic processes. Metabolic liver disorders pose significant health challenges and have historically been difficult to treat. Traditional treatments involve dietary management and enzyme replacement therapies, which can be burdensome and only partially effective. For a long time, liver transplantation was the only curative option for these inherited disorders, requiring lifelong immune suppression. But today, a medical revolution is underway.

Recent breakthroughs in gene therapy and genome editing technologies offer promising avenues for effective and long-lasting treatments. By correcting the underlying genetic defects, these cutting-edge approaches in molecular medicine can restore normal metabolic function and significantly improve the quality of life for affected individuals. What was considered revolutionary just a few years ago, is now being used successfully in humans solving previously very unfavorable conditions. Despite undeniable successes and continuous development of novel approaches, challenges remain to improve safety and efficacy of gene therapies, such as pre-existing humoral response, long-term transgene persistence, potential genotoxicity, or high vector dose-related acute inflammatory responses.

Ongoing research focuses on improving the precision and safety of these therapies. Innovations include the development of novel vectors for efficient gene delivery, high-fidelity endonucleases to minimize off-target effects, and strategies to modulate the immune response to allow for repeated administration. Additionally, there is an increased possibility for translating these therapies into clinical practice due to progresses in vector design, manufacturing and regulatory paths.

The research presented in this Research Topic highlights the transformative potential of these technologies.

The review article proposed by Palacio et al. “*Revolutionizing In vivo Therapy with CRISPR/Cas Genome Editing: Breakthroughs, Opportunities and Challenges*,” delves into the revolutionary impact of CRISPR/Cas9 technology on *in vivo* therapy. It discusses the breakthroughs achieved in precise genome editing, the opportunities for treating a wide range of genetic disorders, and their challenges, such as off-target effects and delivery efficiency.

Diverse strategies to improve adeno-associated virus (AAV) vectors are discussed by Shitika et al. in “*AAV-based Vector Improvements Unrelated to Capsid Protein Modification*.” This research addresses the advancements in AAV-based vector technology, focusing on improvements that do not involve capsid protein modification but modifying the structures determining the viral life cycle. These innovations enhance AAV production, transduction efficiency, and transgene expression in tissues of interest, making gene therapy more effective and reducing the risk of side effects.

Severe metabolic liver disorders and unmet medical needs that are glycogen storage diseases type I (GSDI) and familial hypercholesterolemia (FH), are examples of conditions for which gene therapy and gene editing offer promising therapeutic options for the future.

GSDI are characterized by the accumulation of glycogen in the liver and kidneys due to a deficient glucose-6-phosphatase enzyme activity, leading to impaired glucose release and various metabolic complications. Several gene therapy strategies have been investigated to cure GSDI and they are reviewed here by Chou and Mansfield in the article “*Gene Therapy and Genome Editing for Type I Glycogen Storage Diseases*.” Notably, authors highlight the progress made in AAV-mediated gene augmentation approaches and their current potential to provide a permanent cure for these diseases. They also propose a brief overview of the most recent research using CRISPR/Cas9-based *in vivo* genome editing technology developed for these conditions.

FH is a genetic disorder that affects how the body processes cholesterol, characterized by very high levels of low-density lipoprotein (LDL) cholesterol. This can lead to an increased risk of cardiovascular disease and early heart attacks if not managed properly. Current treatments focus on lowering LDL cholesterol levels, thanks to medications (statins, PCSK9 inhibitors), diet rich in fruits and vegetables, or lipoprotein apheresis. Within the article “*Gene Transfer and Genome Editing for Familial Hypercholesterolemia*,” Canepari and Cantore explore the use of gene transfer and genome editing to correct this disease, discussing their advantages and weaknesses, and outline the strategies that could become the drugs of tomorrow.

Overall, the researches presented in this Research Topic underscores the significant strides made in gene therapy and genome editing for metabolic liver disorders. The collective efforts of the scientific community in advancing these therapies not only pave the way for curing metabolic liver disorders but also set the stage for tackling a broader range of genetic diseases. The journey ahead is dense of opportunities and challenges, but the potential benefits for patients are countless, making this an exciting and pivotal area of research.

